# Eyes with Large Disc Cupping and Normal Intraocular Pressure: Using Optical Coherence Tomography to Discriminate Those With and Without Glaucoma

**Published:** 2014

**Authors:** Tiago S. Prata, Syril Dorairaj, Luisa Trancoso, Fabio N. Kanadani, Luis G. Biteli, Rafael Furlanetto, Flavio S. Lopes, Mauro T. Leite

**Affiliations:** 1 Federal University of São Paulo, Rua Sena Madureira, 1500 - Vila Mariana, São Paulo - SP, 04021-001, Brazil; 2 Glaucoma Unit, Hospital Medicina dos Olhos, Rua Salém Bechara, 297 - Centro, Osasco - SP, 06018-180, Brazil; 3 Department of Ophthalmology, Mayo Clinic, 4500 San Pablo Road, Jacksonville, Florida, 32224, USA

**Keywords:** Diagnostic Ability, Optic Disc, Glaucoma, OCT, SD-OCT

## Abstract

We evaluated the ability of spectral-domain optic coherence tomography (SD-OCT) to differentiate large physiological optic disc cupping (LPC) from glaucomatous cupping in eyes with intraocular pressure (IOP) within the normal range. We prospectively enrolled patients with glaucoma or presumed LPC. Participants had optic discs with confirmed or suspected glaucomatous damage (defined as a vertical cup-to-disc ratio≥0.6), and all eyes had known untreated IOP<21 mmHg. For glaucomatous eyes, a reproducible glaucomatous visual field (VF) defect was required. LPC eyes required normal VF and no evidence of progressive glaucomatous neuropathy (follow-up≥30 months). Peripapillary retinal nerve fiber layer (pRNFL) and macular ganglion cell complex (GCC) thicknesses were obtained using SD-OCT. For all studied parameters of pRNFL and GCC thicknesses, eyes with glaucoma (n=36) had significantly thinner values compared to eyes with LPC (n=71; P<0.05 for all comparisons). In addition, pRNFL parameters had sensitivity of 66.7% and specificity of 83.1%, and GCC parameters had sensitivity of 61.2% and specificity of 81.7%. The combination of the two analyses increased the sensitivity to 80.6%. In conclusion, while evaluating patients with large optic disc cupping and IOP in the statistically normal range, SD-OCT had only limited diagnostic ability to differentiate those with and without glaucoma. Although the diagnostic ability of the pRNFL and the GCC scans were similar, these parameters yielded an increase in sensitivity when combined, suggesting that both parameters could be considered simultaneously in these cases.

## INTRODUCTION

The term glaucoma suspect, advocated by Shaffer (1) in the 1970s, has been used to identify two main populations of individuals or eyes: those with consistently elevated intraocular pressure (IOP; ocular hypertensives) and those whose optic nerve head (ONH) and/or peripapillary retinal nerve fiber layer (pRNFL) appearance are suggestive of, but not definitive for, glaucoma (1-3). Among all glaucoma suspects, eyes with optic nerve features suspicious or suggestive of early glaucoma are probably those that offer the greatest challenge for clinicians. In contrast with the robust longitudinal data published on ocular hypertension (4-7), there is no specific management guideline for patients with suspicious ONH appearance. 

Since the introduction of time-domain optical coherence tomography (TD-OCT), different studies have consistently shown that pRNFL parameters had a better performance compared to total macular thickness for the detection of glaucoma (8-12). With the advent of spectral-domain optical coherence tomography (SD-OCT), a significant improvement in imaging resolution was achieved, allowing segmentation of the macular region and better identification of each layer (13-14). The RTVue SD-OCT (RTVue-100 OCT; Optovue, Inc., Fremont, CA), one of the commercially available SD-OCT devices, provides a segmented evaluation of the macular inner retinal layers. This specific analysis is called ganglion cell complex (GCC) scan, and consists of three layers: the RNFL, ganglion cell layer, and inner plexiform layer (15). Recent studies demonstrated GCC thickness as a useful parameter for early glaucoma diagnosis (16-18).

 Studies evaluating the ability of SD-OCT to detect glaucoma usually include a cohort of healthy individuals versus individuals with established glaucoma and elevated IOP. However, on daily practice, we often deal with eyes with large optic disc cups and IOP within the normal range. In this scenario, it is not an easy task to determine whether a patient has glaucoma or just a large physiological optic disc cup (LPC). In the present study, we sought to evaluate the ability of different SD-OCT parameters (conventional pRNFL and macular GCC scans) to differentiate presumed LPC from glaucomatous cupping in eyes with IOP in the statistically normal range. 

## MATERIALS AND METHODS

*Participants*

In this observational case-control study, participants were recruited from Hospital Medicina dos Olhos (Osasco, Brazil). Written informed consent was obtained from all participants. The Federal University of São Paulo approved all protocols and the methods described followed the tenets of the Declaration of Helsinki.

All participants underwent a comprehensive ophthalmological evaluation, including best-corrected visual acuity, slit-lamp biomicroscopy, IOP measurement, gonioscopy, dilated fundoscopy, visual field testing (VF; standard automated perimetry; Humphrey SITA - Standard 24–2, Carl Zeiss Meditec, Dublin, CA), optic disc stereophotographs and imaging with SD-OCT (RTVue-100 OCT; Optovue, Inc., Fremont, CA).

To be included, individuals with presumed LPC required normal VF testing in both eyes. Included eyes had to have a suspicious appearing optic disc, defined as a vertical cup-to-disc ratio (CDR)≥0.6, and at least 30 months of follow-up with no evidence of progressive optic neuropathy (assessed by serial color stereophotographs performed at least twice a year, with a maximum interval of 6 months) prior to the SD-OCT imaging session. Based on the ISGEO classification, in most studies the VCDR cut-off value used to separate glaucomatous from healthy eyes was usually determined as 0.7 (based on the 97.5 percentile of the CDR distribution for the studied population) (19,20). In the present study, our goal was to separate participants in glaucomatous and suspect eyes, not healthy eyes. Therefore, we adopted a less strict cut-off value (≥0.6), which we considered more clinically relevant, as many eyes with a CDR of 0.6 would be probably classified as suspects on daily practice. Also, they were required to have IOP<21 mmHg during the follow-up period and no previous history of IOP-lowering medications. Glaucomatous eyes had to have untreated IOP<21 mmHg (based on two separate measurements), evidence of glaucomatous optic neuropathy (GON), and reproducible glaucomatous VF defects. Indices for VF test reliability were set at fixation loss <20%, false-negative <33% and false-positive <15%. Because established glaucoma requires treatment, eyes with glaucoma were not followed over time and imaging was performed at the time of diagnosis. Exclusion criteria for both groups were previous ocular surgery or trauma, spherical equivalent>±6.0 D, use of oral or topical steroids, use of oral medications that could affect IOP (such as oral beta-blockers) and ocular diseases other than glaucoma. 

Characteristic GON was defined as a vertical CDR≥0.6, asymmetry of CDR≥0.2 between eyes, presence of localized pRNFL defects, and/or neuroretinal rim defects in the absence of any other abnormalities that could explain such findings. Two experienced graders evaluated all stereophotographs. In case of disagreement, a third grader was used to adjudicate. A glaucomatous VF defect in the standard automated perimetry was defined as three or more points in clusters with a probability of <5% (excluding those on the edge of the field or directly above and below the blind spot) on the pattern deviation plot, a pattern standard deviation index with a probability of <5%, or a glaucoma hemifield test with results outside the normal limits.

*Procedures*

 Baseline data assessed were age, gender, self-reported race, and IOP (Goldmann applanation tonometry). All patients underwent macular GCC thickness and pRNFL thickness (ONH map) measurement with the RTVue SD-OCT (software version A4). Briefly, the instrument is able to measure the thickness of the retina by using a superluminescent diode light with a center wavelength of 840nm. The GCC scan covered a 7x7 mm scan area centered on the fovea. Global average, superior sector, and inferior sector thicknesses for the two scan protocols were used for analysis. Images with signal strength index less than 40 or not well-centered (subjective assessment) were excluded from the analysis. All images were acquired by two experienced operators (one from each center) who were masked to patient’s clinical data. 

*Statistical Analysis*

Descriptive statistics included mean and standard deviation for normally distributed variables and median and quartiles for non-normally distributed variables. To evaluate the ability of the SD-OCT to detect eyes with glaucoma, receiver operating characteristic (ROC) curves were built and the areas under ROC (AUC) calculated. Because of the potential influence of age on the diagnostic ability of the SD-OCT, an ROC regression method was performed using age as a covariate. The pairwise comparison of the AUCs obtained for each parameter was performed using a method proposed by Pepe et al. (21). To account for the potential correlation between eyes, the cluster of data for the study subject was considered as the unit of resampling when calculating standard errors. In addition, we evaluated the performance of the RTUue-100 OCT normative database in depicting statistically abnormal results. Eyes were considered abnormal if they had at least two borderline sectors (p<0.05, color-coded in yellow) or one abnormal sector (p<0.01%, color-coded in red) on either pRNFL or GCC analyses (average, superior or inferior regions). To evaluate the overall performance of pRNFL combined with GCC, two distinct criteria were employed. For the first criterion, eyes were considered abnormal if either pRNFL and/or GCC were abnormal (focusing on sensitivity). For the second, eyes were considered abnormal if pRNFL and GCC were abnormal (focusing on specificity). All statistical analysis was performed using Stata (Stata version 10; StataCorp, College Station, Texas, USA). The alpha level (type I error) was set at 0.05.

## RESULTS

A total of 36 eyes from 23 glaucomatous patients and 71 eyes from 38 individuals with presumed LPC were included. Glaucomatous patients were on average older (52.5 vs 41.7yo; p=0.004) compared to individuals with LPC. The median VF mean deviation (MD) and pattern standard deviation (PSD) in the glaucoma group were -2.7dB and 2.3dB, respectively, indicating an early VF loss. Table 1 provides additional clinical and demographic characteristics of included eyes. 

Table 2 shows the comparison between pRNFL and GCC thicknesses in individuals with LPC and glaucoma. For all studied parameters, eyes with glaucoma had significantly thinner values compared to eyes with LPC (P<0.05 for all comparisons). 

**Figure 1:**
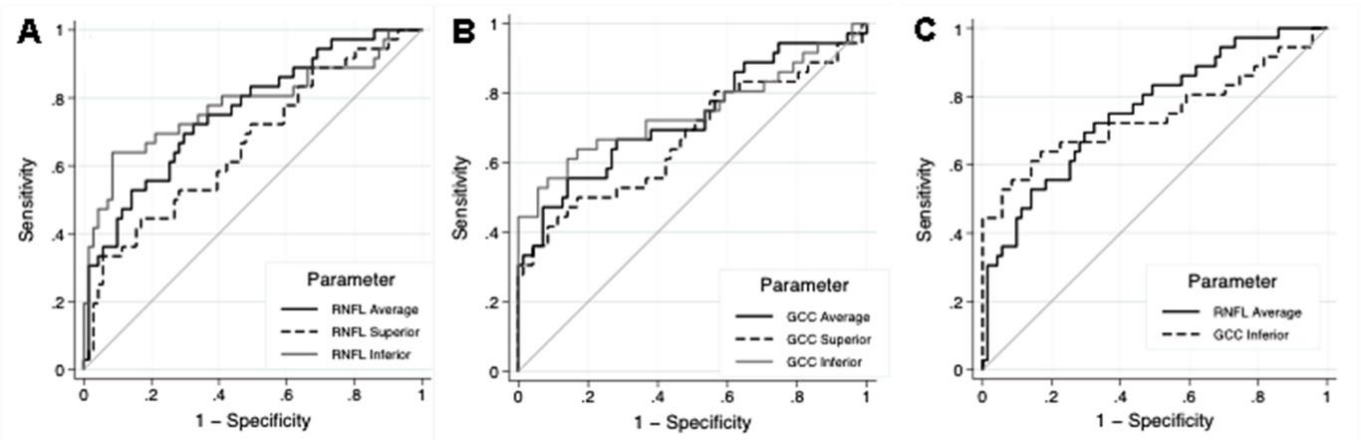
Age-adjusted receiver operating characteristics (ROC) curves for the average, inferior and superior parapapillary retinal nerve fiber layer (RNFL) thickness (A) and ganglion cell complex (GCC) thickness (B) obtained with the RTVue SD-OCT. Comparison of ROC curves between the best RNFL (average thickness) and GCC (inferior thickness) parameters (C).

Regarding the performance of each SD-OCT parameter, the pRNFL thickness parameter with the largest AUC was the average pRNFL thickness (0.758) followed by the inferior (0.744) and superior sectors (0.663; Fig. 1A). The GCC thickness parameter with the largest AUC was the inferior sector (0.762), followed by the average (0.730) and superior sector (0.672; Fig. 1B). No significant difference was found between the parameters with larger AUCs from pRNFL and GCC analyses (p=0.87; Fig. 1C). Based on the comparison between ROC curves in Fig. 1C, the GCC analysis had a better performance in the first half of the graphic (better specificity, but worse sensitivity), while the pRNFL analysis had a better performance in the second half of the graphic (better sensitivity, but worse specificity).

**Figure 2:**
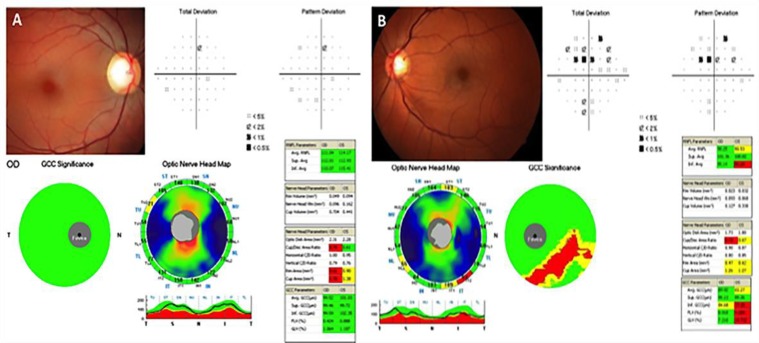
Patient with large physiological optic disc cup and intraocular pressure within the normal range (right eye) followed for 4 years without any signs of progressive optic neuropathy. Note that all RNFL and GCC parameters are within the normal range (A). Patient with glaucomatous cupping and intraocular pressure within the normal range (left eye). Note the inferior RNFL defect associated with superior visual field loss and abnormal RNFL and GCC parameters (B).

 Using the normative database, the pRNFL analysis had a sensitivity of 66.7% and a specificity of 83.1% while the GCC analysis had a sensitivity of 62.9% and a specificity of 80.6%. Considering either abnormal pRNFL or GCC parameters as glaucoma yielded an increased in sensitivity to 80.6% at the cost of specificity (74.6%). Considering pRNFL and GCC parameters as glaucomatous resulted in an increase in specificity to 90.1% at the cost of sensitivity (47.2%). Examples of SD-OCT results in patients with LPC and glaucomatous cupping are given in Fig. 2A and 2B, respectively.

**Table 1:** Demographic and Ocular Characteristics of Study patients

**Parameter**	**LPC (n=71)**	**Glaucoma (n=36)**	**P value**
**Age (y)**	41.7 14.5	52.512.9	0.004
**Sex (% male)**	39	30	0.57
**MD (dB) ** *	-1.01 (-1,61, -0.2)	-2.66 (-4.67, -1.65)	<0.001
**PSD (dB) ** *	1.46 (1.28, 1.86)	2.32 (2.03, 3.84)	<0.001

LPC: Presumed large physiological cupping; MD: mean deviation; PSD: pattern standard deviation.

* Non-normally distributed variables; represented by median (first quartile, third quartile).

**Table 2:** Comparison between optic coherence tomography parameters in eyes with presumed large physiologic cupping (LPC) and eyes with glaucoma

**Parameters ** *	**LPC (n=71)**	**Glaucoma (n=36)**	**P value**
**RNFL Avg (m)**	103.259.04	92.2910.20	<0.001
**RNFL Sup (m)**	102.2311.12	93.4812.62	0.012
**RNFL Inf (m)**	104.258.57	91.0511.82	<0.001
**GCC Avg (m)**	91.915.59	84.1010.37	0.005
**GCC Sup (m)**	91.475.98	84.8610.78	0.02
**GCC Inf (m)**	92.375.56	83.3411.65	0.003

RNFL: Retinal nerve fiber layer; GCC: Ganglion cell complex; Avg: average; Inf: Inferior; Sup: Superior.

* Data presented as mean   standard deviation**.**

## DISCUSSION

 The present study demonstrated that the RTVue SD-OCT was able to discriminate glaucomatous eyes from eyes with suspicious-appearing optic discs. In addition, we demonstrated that although the GCC and pRNFL scans had a similar performance to detect glaucoma, these parameters yielded an increase in sensitivity when combined. To the best of our knowledge, this is the first study to evaluate the performance of RTVue SD-OCT for the detection of glaucoma in a population of suspects. Our results may provide new information on the use of SD-OCT on a clinically relevant population.

 The inexistence of a perfect standard for glaucoma diagnosis has limited the study of imaging instruments on glaucoma suspects. For that reason, most studies have focused on the ability of these instruments to discriminate between eyes with established glaucomatous VF damage and healthy individuals, leading to an overestimation of their performance.22-27 Although a common situation on a clinical scenario, there is scant information in the literature on the diagnostic abilities of these instruments for glaucoma suspects with large optic disc cups and IOP within the normal range. In the present study, we sought to investigate the ability of the SD-OCT to distinguish between glaucoma and non-glaucomatous eyes with suspicious appearing optic disc. Therefore, we included eyes with no observed progression on optic disc over time as our control group. Medeiros et al. (27) previously suggested a similar approach. In their study, the authors found that the diagnostic accuracy of an imaging device (confocal scanning laser ophthalmoscopy more specifically) in glaucoma could vary significantly depending on the reference standard used to define study patients and controls. The authors concluded that data derived from case-control studies including well-defined groups of subjects with or without disease might not be applicable to the clinically relevant population. 

 Overall, our diagnostic accuracies were lower compared to previous reports using SD-OCT technology (8,16,24,25). For example, in eyes with early glaucoma, Rao et al reported AUCs of 0.82 for the inferior pRNFL thickness (28). For the detection of normal tension glaucoma, Kim et al reported AUC of 0.85 for the inferior pRNFL (29). In the present study, for the same parameter, we obtained a lower AUC of 0.74. This worse performance was expected since our glaucomatous patients had early VF damage (26) and our control group consisted of eyes with suspicious-appearance of the optic disc, illustrating the influence of the control group on the diagnostic performance of the test. We believe this type of investigation is more clinically relevant as it resembles what happens in daily practice. In fact, using the GDx to detect glaucoma in a population of pre-perimetric glaucoma, Medeiros et al found AUCs of 0.78 for the average thickness parameter, similar to 0.76 found for the average pRNFL in the present study (30). 

 Conventional analysis of the pRNFL is a widely used tool in glaucoma diagnosis (22,24,25,31-33). On the other hand, macular thickness measurement by TD-OCT has not been frequently used due to poor diagnostic performance (8,34). The role of examining the macular region in glaucoma has been supported by the fact that structural damage in glaucoma occurs primarily in the RGCs layer, which is denser in the macular region (35). However, segmented evaluation of the macular inner retinal layers was only feasible with the advent of SD-OCT imaging. In this context, there are several studies comparing GCC and pRNFL protocols for glaucoma diagnosis (36-38). In a recent study, Rao et al found that the GCC outperformed the pRNFL thickness to detect early glaucoma (36). However, the sensitivities of these parameters at high specificity (95%) were comparable (52.7% vs 58.2%). Evaluating patients with normal-tension glaucoma, Seong et al found that the GCC scan had a diagnostic ability comparable to that of the pRNFL scan in patients with early VF defects (15). Our group has recently reported on the diagnostic performance of these two analyses (18). We found that the GCC scan had a similar or even slightly superior ability to discriminate between eyes with early glaucoma and controls when compared to the pRNFL scan. In the present study, we evaluated a different and more specific population, as we included patients with LPC (vs early glaucoma) instead of healthy controls as the other cited studies did. Notwithstanding, our results are in agreement with these previously published data, in a sense, because pRNFL and GCC scans showed similar diagnostic performances. 

 In a clinical setting, ancillary tests should be accurate and easy to interpret. Several studies have evaluated the diagnostic performance of imaging instruments in ophthalmology using ROC curves. However, translating this information to the clinical scenario can be difficult. Therefore, the ability of the normative database for the detection of glaucoma was evaluated for each scan separately and in combination. Two criteria were used for the combined parameters. In the first, the exam was said to be abnormal if either pRNFL or GCC were abnormal. The second criterion required both abnormal scans for the exam to be considered abnormal. Using the first criterion, a significant increase in sensitivity was found, albeit a small decrease in specificity. Using the second criterion, a significant increase in specificity was found; however, the decrease in sensitivity might have made the criteria too strict to be considered in clinical practice (e.g. many patients with glaucoma would be labeled as healthy). Nevertheless, our findings suggest that each scan is detecting glaucoma in different patients and may improve their accuracy if used in conjunction. 

 It is important to stress some specific characteristics and limitations of the present study. First, we defined IOP within the normal range without considering diurnal IOP variation and central cornea thickness influence on applanation tonometry measurements. Second, glaucomatous patients were, on average, older than patients with LPC, thus introducing a potential confounding factor. To minimize the effect of age on our results, the ROC model was corrected by age, as proposed by Pepe et al. (21). Third, it is possible that some eyes with presumed LPC will develop glaucomatous progression over time. By including LPC eyes with at least 30 months of follow-up without progression, we expect to reduce this occurrence. Fourth, we did not investigate the correlation between disease severity and SD-OCT diagnostic performance because our study population had a narrow range of disease severity (most patients had early glaucoma) and relatively small sample size. Finally, although patients may demonstrate early structural changes in the optic nerve or RNFL without any VF defect, some patients have shown evidence of functional deterioration without measurable changes in their scores on currently available structural tests. It highlights the importance of combining this proposed structural assessment with functional tests on daily practice. These limitations should be considered while interpreting our results.

 In conclusion, while evaluating patients with large optic disc cupping and IOP in the statistically normal range, SD-OCT had only limited diagnostic ability to differentiate those with and without glaucoma. Although the diagnostic ability of the pRNFL and the GCC scans were similar, these parameters yielded an increase in sensitivity when combined, suggesting that both parameters could be considered simultaneously in these cases.
